# NLRP3 Inflammasome and Inflammatory Bowel Disease

**DOI:** 10.3389/fimmu.2019.00276

**Published:** 2019-02-28

**Authors:** Yu Zhen, Hu Zhang

**Affiliations:** ^1^Department of Gastroenterology, West China Hospital, Sichuan University, Chengdu, China; ^2^The Centre of Inflammatory Bowel Disease, West China Hospital, Sichuan University, Chengdu, China

**Keywords:** NLRP3 inflammasome, mucosal immune, gut homeostasis, ulcerative colitis, Crohn's disease

## Abstract

NLRP3 inflammasome can be widely found in epithelial cells and immune cells. The NOD-like receptors (NLRs) family member NLRP3 contains a central nucleotide-binding and oligomerization (NACHT) domain which facilitates self-oligomerization and has ATPase activity. The C-terminal conserves a leucine-rich repeats (LRRs) domain which can modulate NLRP3 activity and sense endogenous alarmins and microbial ligands. In contrast, the N-terminal pyrin domain (PYD) can account for homotypic interactions with the adaptor protein-ASC of NLRP3 inflammasome. These characters enable it function in innate immunity. Its downstream effector proteins include caspase-1 and IL-1β etc. which exhibit protective or detrimental roles in mucosal immunity in different studies. Here, we comprehensively review the current literature regarding the physiology of NLRP3 inflammasome and its potential roles in the pathogenesis of IBD. We also discuss about the complex interactions among the NLRP3 inflammasome, mucosal immune response, and gut homeostasis as found in experimental models and IBD patients.

## Introduction

Inflammatory bowel disease (IBD), comprising Crohn's disease (CD) and ulcerative colitis (UC), is characterized by chronic and relapsing inflammation in the gastrointestinal tract, and some hypotheses propose that damage to the intestinal mucosa occurs as a result of dysregulated innate immune response (1). Hence, understanding the regulatory circuits that control aberrant innate immune responses in the intestine is critical to elucidate the pathogenesis of IBD. The NLRs family member NLRP3 is rapidly emerging as a crucial regulator of intestinal homeostasis. This innate immune receptor mediates the assembly of the inflammasome complex in the presence of microbial ligands, triggering activation of caspase-1 and secretion of interleukin-1β (IL-1β) and IL-18, and has been implicated in the pathogenesis of IBD (2), but the detailed role of NLRP3 inflammasome in IBD is still debated. Early studies reported that NLRP3 inflammasome-induced production of IL-1β and IL-18 contributed to intestinal inflammation. However, the concept of detrimental inflammasome signaling in IBD is being re-evaluated due to recent reports that IL-1β and IL-18 production can confer protection against colitis. Here, we comprehensively review the current literature regarding the physiology of NLRP3 inflammasome in the intestinal, and we also discuss about the complex interactions among the NLRP3 inflammasome, mucosal immune response and gut homeostasis as found in experimental models and IBD patients.

## Definitions and Composition of NLRP3 Inflammasome

Inflammasomes are a group of cytosolic protein complexes that can recognize various stress, exogenous microbes, and endogenous danger signals. Inflammasomes respond to them by activating caspase-1, producing IL-1β, and IL-18, and starting the inflammatory process (3, 4). The definition of inflammasome was first made by the group of Tschopp in 2002 (5), they found that it played a critical role in microbial infections and also in the regulation of both mucosal immune responses and metabolic processes. Inflammasomes comprise several subtypes, among which the NLRP3 inflammasome is one of the most studied. The multiprotein complex of NLRP3 inflammasome consists of the sensors-NLRP3, the adaptor-apoptosis-associated speck-like protein containing a caspase recruitment domain (ASC) and the effector protein-caspase-1 (6), and it can be widely found in immune cells including granulocytes, antigen presenting cells (APC), macrophages, T and B lymphocytes (6). As the core protein of NLRP3 inflammasome, NLRP3 contains a central nucleotide-binding and oligomerization (NACHT) domain which facilitates self-oligomerization and has ATPase activity. The C-terminal conserved LRRs domain can modulate NLRP3 activity and sense endogenous alarmins and microbial ligands. In contrast, the N-terminal pyrin domain (PYD) can account for homotypic interactions with the adaptor protein-ASC. ASC contains two transduction domains, one is a pyrin domain which can connect the upstream NLRP3, the other is a caspase recruitment domain (CARD) which can connect the downstream caspase-1 (7). As the effector protein of NLRP3 inflammasome, caspase-1 can convert pro-IL-1β and pro-IL-18 into their active forms-IL-1β and IL-18. In addition, caspase-1 is also one of the essential factors involved in an inflammatory form of cell death termed pyroptosis (8). In summary, the components of NLRP3 inflammasome enable it function as an immunological player after activation.

## Activation and Regulation of NLRP3 Inflammasome

**Figure 1:**
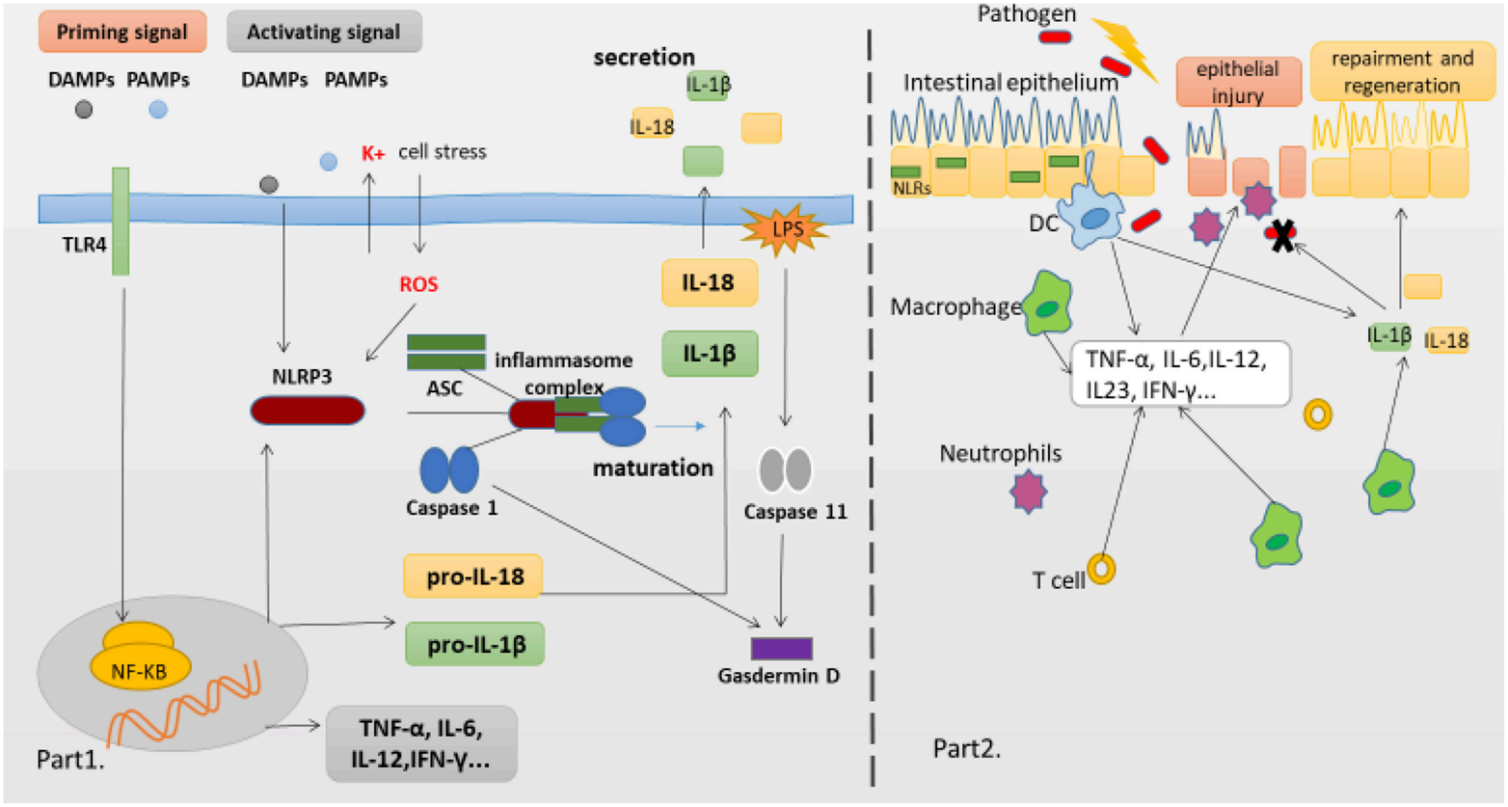
NLRP3 Inflammasome complex and pathology of the IBD. **(Part 1)** The production of proinflammatory cytokines IL-1β and IL-18 is a two-step process. NF-κB activation can rapidly prime the expressions of pro-IL-1β and pro-IL-18, which are then cleaved into the mature IL-1β and IL-18 by caspase-1 in the Inflammasome. The second signal can directly activate inflammasome assembly. And cleavage of gasdermin D by caspase-11 and caspase-1 is essential for pyroptosis of innate immune cells and endothelial cells harboring LPS-tainted cytoplasm. **(Part 2)** The intestinal epithelial barrier protects underlying mucosal tissues from commensal bacteria present in the gut. But in susceptible hosts, the epithelial barrier is compromised allowing commensal bacteria to invade lamina propria and mucosa. Infiltrated bacteria interact with macrophages, Dendritic cells(DC) and neutrophils via innate recognition receptors such as NLRs. Activation of NLRs induces the production of proinflammatory cytokines which further recruit immune cells to the infected tissue accelerating inflammatory response.

The innate immune system is the first line defense, which can sense microbes or endogenous danger signals via recognition of damage-associated molecular patterns (DAMPs) or pathogen-associated molecular patterns (PAMPs) by host pattern recognition receptors (PRRs), such as Toll-like receptors (TLRs) and NLRs. The NLRs family member NLRP3 is essential for the recognition of PAMPs or DAMPs. NLRP3 inflammasome plays a critical role in inflammatory response as a major component of innate immunity, it provides a molecular platform which can be activated by multiple endogenous and exogenous stimuli including ATP, microbial agonists, particulate matters and pore-forming toxins (9–11). When the basal level of NLRP3 expression is competent for inflammasome activation in resting cells, a two-step process is required (Part 1 of Figure 1). The first or priming signal converges on the activation of nuclear factor kappa-B (NF-κB) and transcriptional induction of NLRP3 itself and pro-IL-1β. The second or activating signal, which may be a microbial or danger signal, can directly activate inflammasome assembly (12). Some mechanisms have been proposed to elucidate the activation of NLRP3 inflammasome, including K+ efflux (13), cathepsin B leakage from lysosomes (14), reactive oxygen species(ROS) production (15), translocation to the mitochondria (16), and mitochondrial dysfunction (17), despite none of them has been found to be unified for all stimuli. In addition, NLRP3 inflammasome activation also occurs downstream of caspase-11 and gasdermin D cleavage and pore-formation in a process called non-canonical inflammasome activation (18, 19). But whether and how priming signal affects inflammasome assembly and subsequent activation have remained elusive, newer data are claiming that the synthesis of mitochondrial DNA (mtDNA), induced after the engagement of Toll-like receptors, is crucial for NLRP3 signaling. Toll-like receptors signal can function via the MyD88 and TRIF adaptors to trigger IRF1-dependent transcription of CMPK2 (a rate-limiting enzyme that supplies deoxyribonucleotides for mtDNA synthesis) (20). However, none of these hypotheses can fully explain the activation process of NLRP3 inflammasome, it is possible that these hypotheses may all coexist in this process. Membrane permeation, ROS production and mitochondrial dysfunction are all interrelated cellular events, and then more studies are warranted to elucidate the exact mechanisms.

Although the activation of NLRP3 inflammasome is largely beneficial to the host defense during infections and metabolic processes, over production of IL-1β and IL-18 results in sterile inflammation, which can increase the risk of developing metabolic and autoinflammatory diseases among patients. Therefore, in order to avoid overt tissue damage, the activation of NLRP3 inflammasome must be finely controlled. Both scaffolding proteins and post-translational modifications are responsible for these fine regulations, and then they tightly control and modulate the NLRP3 inflammasome activation together. Actually, NF-κB- induced transcription regulates the expression of NLRP3 inflammasome which further requires stimulus from cytokine-signaling pathways or sensitization by a TLR or CLR ligand (21). Although caspase-8 can suppress NLRP3 activities in dendritic cell (DC) (22), it contributes to a robust activation of NF-κB due to TLR stimulation in macrophages, T and B lymphocytes, and even natural killer (NK) cells (23–26). The evidence reminds us that the negative effect of caspase-8 on the NLRP3 inflammasome may be specific to DC. The canonical NLRP3 inflammasome activation requires IL-1R–associated kinases (IRAK) and their corresponding kinase activities. A recent study, however, has identified a “priming-independent” mode named as the transcription-dependent activation, which occurs independently of IRAK1 and IRAK4 in lipopolysaccharide (LPS)-primed cells followed by ATP treatment (27). Post-translational modifications of NLRP3, including ubiquitination and deubiquitination, can also either suppress or activate inflammasome activation (28). Recently, Yan et al. reported that Omega-3 fatty acids repressed NLRP3 inflammasome activation and inhibited subsequent caspase-1 activation and IL-1β secretion via G protein-coupled receptor 120 (GPR120) and GPR40 (28). Soon afterwards, their data demonstrated that neurotransmitter dopamine (DA) could also inhibit NLRP3 inflammasome activation through dopamine D1 receptor (DRD1), suggesting DRD1 signaling could negatively regulate NLRP3 inflammasome via a second messenger cyclic adenosine monophosphate (cAMP), which binds to NLRP3 and promotes its ubiquitination and degradation (29). Moreover, it has been indicated that IL-10 can function as a critical transcriptional and post-translational regulator of NLRP3 inflammasome activation in both murine and human macrophages by controlling intestinal inflammation and maintaining gut homeostasis (30). It is worth noting that non-canonical maturation of IL-1β can occur via the NLRP3 inflammasome and caspase-11 during acute inflammatory conditions (31), and several studies have found the role of caspase-11 in mediating expression of IL-1β and IL-18. In particular, a caspase-11-dependency for the production of IL-1β and IL-18 is showed in intestinal tissues harvested from DSS-induced mice (32, 33). Additionally, Shenoy et al. (34) reported that guanylate binding protein 5 (GBP5) could enhance the NLRP3 inflammasome assembly in response to bacterial and bacterial cell wall components but not crystalline agents. Another key regulator of NLRP3 inflammasome activation is NEK7 (NIMA-related kinase 7), which is involved in the regulation of the cell cycle, mitotic spindle formation and cytokinesis. Interaction between the NLRP3 LRR domain and NEK7 leads to NLRP3 inflammasome activation in interphase cells downstream of potassium efflux independent of its kinase activity (35). Lately, Lang et al. (36) reported that inhibition of macrophage migration inhibitory factor (MIF) regulated the release of IL-1α, IL-1β, and IL-18, not by affecting transcription or translation of these cytokines, but via activation of NLRP3 inflammasome. As discussed above, activation and regulation of NLRP3 inflammasome have been increasingly appreciated, it is intriguing for researchers to explore the roles played by NLRP3 inflammasome in IBD which is a common autoimmune disease in the GUT.

## ROS in IBD

ROS refers to a class of special oxygen-containing compounds that have much higher chemical activity than the oxygen. In normal condition, a basal level of ROS has bactericidal effects, participating in the intestinal defensive function. However, in the process of chronic inflammation (such as IBD), excessive ROS produced by the infiltrated neutrophil can trigger oxidative stress (OS) and proteolytic enzymes, which act on endothelial cells and cause cell injury and subsequent intestinal mucosal barrier damage and luminal pathogen invasion, and further in turn exaggerate inflammatory cell infiltration and inflammatory damage, eventually leading to intestinal mucosal necrosis and ulceration (37). Besides, accumulated ROS could also act as secondary chemical messengers for the activation of intracellular signal pathways, such as p38 mitogen-activated protein kinase (MAPK), and NF-κB, to influence cell proliferation, differentiation and apoptosis (38, 39). As transcription factors, a deregulation of NF-κB, signaling, such as oxidative activation, enhances expression of various proinflammatory cytokines in the intestinal epithelial cells, such as tumor necrosis factor alpha (TNF-α), IL-1, IL-8, and facilitates inflammation and carcinogenesis (40). MAPKs are highly conserved serine/threonine protein kinases which can function in various cellular processes, such as proliferation, differentiation, and apoptosis, as well as stress response (41). In UC tissues, p38MAPK signaling changes are a molecular signature of UC and proportional to the degree of inflammation (42). Recently Zhong et al. (43) suggested that particulate stimuli might induce ROS production in mitochondria, and further trigger a calcium influx mediated via transient receptor potential melastatin 2 (TRPM2), resulting in an activation of the NLRP3 inflammasome. While many activators of the NLRP3 inflammasome generate ROS, the interruption of ROS production with pharmacological inhibitors can block the activation of NLRP3 (44). These data indicate that the generation of ROS which is a critical factor in mucosal immunity, may be an essential upstream event for NLRP3 inflammasome activation.

## NLRP3 Inflammasome in Mucosal Immune Response

Although the precise etiology of IBD remains unclear, aberrant immune responses against commensal microflora are widely thought to underlie IBD (1, 45). Furthermore, it is worth noting that chronic inflammation in patients with IBD is associated with alterations in adaptive immune responses represented by a Th2 profile in UC patients and a Th1/Th17 profile in CD patients (46, 47). The innate immune system is critical to control host resistance, one pivotal player within this system is PRRs, which can not only translate danger and microbial sensing into immediate host defenses, but also provide a signal to prime the adaptive immune response for further protection. The NLR family member NLRP3 has the ability to facilitate the formation of inflammasome and the activation of MAPK and NF-κB signaling cascades, and then initiate and support robust immune responses. Both MAPK and NF-κB pathways culminate in the transcriptional activation of genes encoding chemokines and cytokines that activate the innate and adaptive immune systems. Recent findings (48) have indicated that IL-1β, but not IL-18, is the most likely effector molecule directly downstream of the NLRP3 inflammasome in the intestine, but IL-18 might be indirectly affected by NLRP3 through secondary effects, because IL-18 deficiency can also abolish the protective effect of NLRP3 in the intestine. Besides, NLRP3 inflammasome further initiates pyroptosis via an activating cleavage of gasdermin D (Gsdmd), which forms pores in the plasma membrane and acts as the executioner molecule for pyroptosis.

### IL-1β

IL-1β is mainly produced by innate immune cells (such as monocytes, DCs, and macrophages), and the major source of IL-1β in colon is macrophages located in the lamina propria (49). During infection, mucosal injury and stress, the activation of IL-1β can trigger local mucosal immune responses, by stimulating T cell proliferation, and direct neutrophils to injury or infection site through the combination of IL-1β and IL-1R complexes (21, 50), and further activate NF-κB and MAPK pathways, leading to the upregulation of other pro-inflammatory cytokines and chemokines (such as IL-6, IL-8, and TNF). Meanwhile, IL-1β can upregulate IL-2 receptor expression, prolonging survival of T cells, and enhance antibody production by B cell proliferation. Early reports showed an overproduction of IL-1β in patients with IBD and mice models, indicating that the function of IL-1β in the development of mucosal inflammation (51, 52). However, recently, many researches in the chemical-induced model have reported that IL-1β can protect mice from intestinal infection of *Citrobacter rodentium* and *Clostridium difficile*, by promoting phagocytosis and eradication of bacteria in mononuclear Phagocytes (53, 54). Besides, a study by Fan et al. (55) also showed that the transplantation of mesenchymal stem cells (MSC) primed by IL-1β could alleviate the chemical-induced colitis. Taken together, the rather ambiguous results concerning the role of IL-1β in mucosal immune response and IBD demand a further investigation with careful consideration.

### IL-18

IL-18 is a multifunctional cytokine, which is mainly expressed in the gut epithelium in both mice and Humans (49), recent findings suggested that the epithelium IL-18 secretion was not dependent on NLRP3 (56), but on caspase-1 (48). However, we know NLRP3 plays a central role in the activation of caspase-1, and then NLRP3 may also contribute to IL-18 production in intestine. IL-18 is functionally found to induce interferon (IFN)-γ and promote Th1 response (57, 58). Early report indicated that IL-18 was upregulated in IBD patients (especially in CD), and had the pro-inflammatory effect of IL-18 by upregulating pro-inflammatory cytokines, such as TNF-α, IL-1, and IL-6 (59, 60), but these studies failed to tell whether the increased IL-18 level in patients was a consequence or causing factor for IBD. Later, polymorphisms in IL-18 genomic locus were showed to be a risk factor for IBD (61). Recently, a series of studies have pointed out that IL-18 can provide protection against colitis and/or colitis-associated cancer (33, 62–66), and IL-18 deficiency may predispose the host to chemically induced colitis. Moreover, IL-18 can induce Th1 cells and NK cell to secrete IFN-γ, which can regulate a proliferation and repairment response in the intestinal tract when the epithelium is injured. And recent studies have also shown that the signals of IFN-γ and downstream STAT-1 were decreased in mice deficient in NLRP3, which were dependent on IL-18 (67). These data indicate that IL-18 may be involved in repair of the epithelial layer of the gut by maintaining proper levels of epithelial cell proliferation during acute experimental colitis. Further, IL-18 also has the function of immunomodulation, for instance, it can enhance proliferation of Th1 cells and host defense against pathogens, inhibit IgE production, and has antitumor effects.

### IL-1α

IL-1α cleavage can be induced by NLRP3 inflammasome stimuli such as nigericin or uric acid crystal, resulting in the co-secretion of both IL-1α and IL-1β. Precursor IL-1α on the surface of several cells, particularly on monocytes and B cells, is referred to as membrane IL-1α. Mice deficient in IL-1α exhibit a reduced inflammation, in which cell death and intracellular IL-1α release do not take place (68). Intrinsic IFN-γ activities depend largely on constitutively expressed IL-1α. Besides, IL-1α also works as a co-stimulator of T cell functions, primarily together with an antigen or a mitogen. It is suggested that IL-1α could contribute to Th2 polarization in mice model (69).

### Pyroptosis

Pyroptosis is a special form of programmed inflammatory cell rupture, which plays a critical role in anti-bacterial innate immune defense and lethal endotoxemia (70). Initially, it's found that pyroptosis was promoted following the activation of pro-caspase-1 by some pathogens, could destroy the infected immune cell and expose the surviving/bacteria to circulating phagocytes and neutrophils, these measures could halt the intracellular replication of pathogens (31, 71). In a spontaneous colitis model deficient in both TLR2 and MDR1A, the activation of inflammasome by commensal bacteria caused myeloid CD11b+ cells to undergo pyroptosis, moreover, similar situation was observed in genetically relevant patients with IBD (72), which suggested that pyroptosis might be involved in the pathogenesis of IBD. Recent findings suggest that cleavage of Gsdmd by mouse caspase-11 or human caspase-4 is also essential for the pyroptosis of innate immune cells and endothelial cells harboring LPS-tainted cytoplasm. Moreover, cleaved Gsdmd also triggers NLRP3-dependent activation of caspase-1 through a cell-intrinsic pathway (19). Mechanistically, Gsdmd is a generic substrate for inflammatory caspases (caspase-1 and caspase-4/5/11), and cleavage of Gsdmd critically determines pyroptosis by releasing the cleaved gasdermin-N domain that bears intrinsic pyroptosis-inducing activity (73). Thus, Gsdmd is revealed as an unexpected, but critical aspect of the anti-bacterial response.

It is well-known that the activation of NLRP3 inflammasome plays an important role in mucosal immune system, however, the disturbed mucosal immunity may lead to the development of auto-immune and inflammatory diseases. We speculate that during the early stage of an inflammatory condition in the gut, NLRP3 may still only engage IL-1β for downstream signaling. But during chronic colitis, NLRP3 in infiltrated myeloid cells may also contribute to IL-18 production. Although studies on IL-18 and IL-1β have found some different results, the effect of these cytokines in repair and restitution of the ulcerated epithelium seems to come to light recently. In addition, pyroptosis has been classified as a defense mechanism of the innate immune system, and the cleavage of new gasdermin family-Gsdmd by inflammatory caspases has also changed our understanding of pyroptosis and programmed necrosis. More studies are warranted to comprehensively explore the functions of these effectors in IBD.

## NLRP3 Inflammasome in gut Homeostasis and IBD

The gastrointestinal environment is a continuous system, which provides energy to the human body and aids in the elimination of waste material. In addition, it prevents infection by providing a vast array of immune cells within the mucosa of GI tract targeting environment toxins and potential pathogens. Accumulating evidence suggests that innate immune recognition at mucosal surfaces particularly within the intestine is an important mediator of intestinal homeostasis (74). Recent studies have highlighted the role for NLRP3 inflammasome, not only as being a crucial mediator of host defense but also being a crucial regulator of gut homeostasis by controlling integrity of the intestinal epithelium and modulating immune responses to microbiota in the gut. However, researches on NLRP3 inflammasome and IBD reported controversial data, most of these studies adopted animal models and some used IBD patients.

### Human Studies

**Figure 2:**
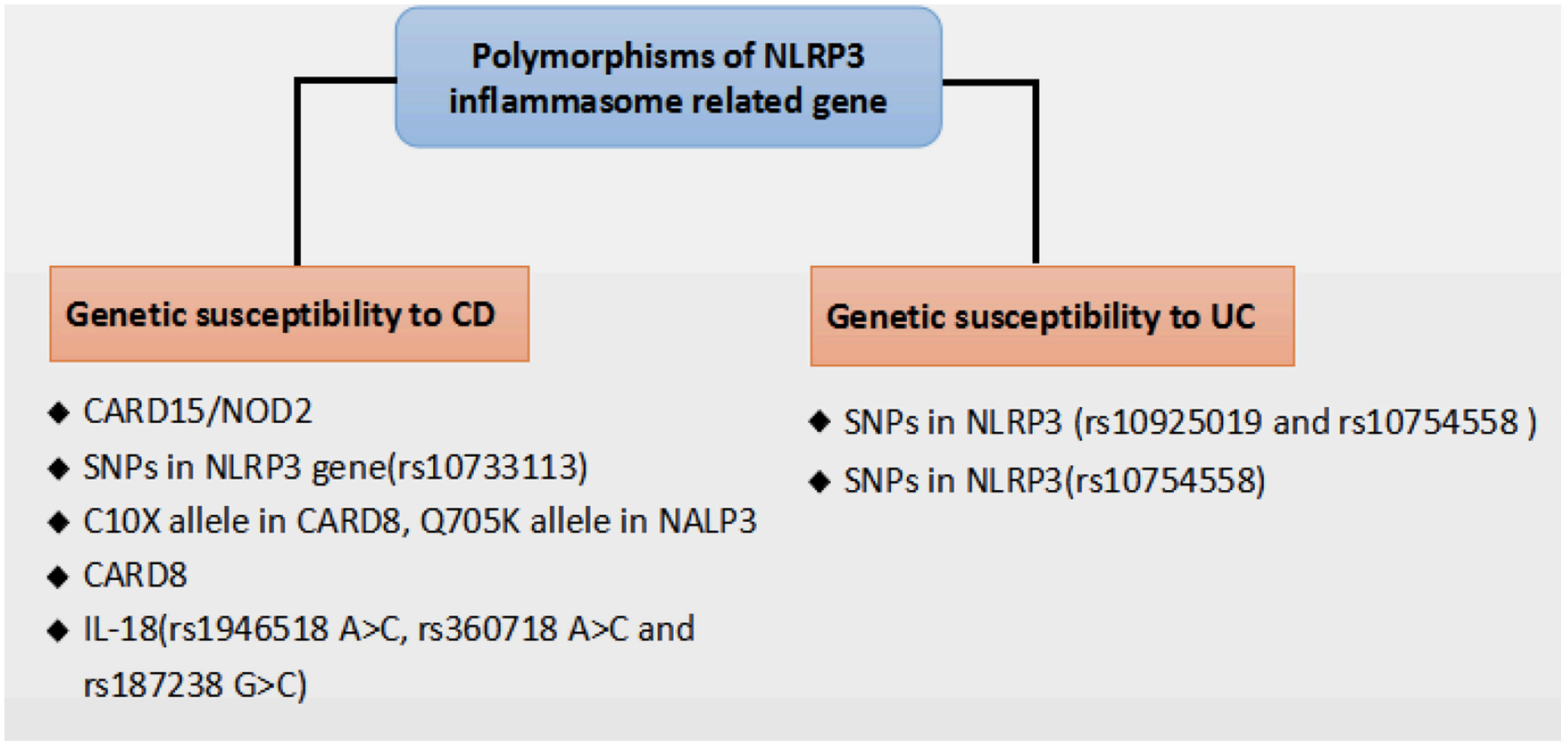
Polymorphisms of NLRP3 inflammasome related gene that affect genetic susceptibility to IBD.

Recent genome-wide association studies have found that polymorphisms conferring a hypofunctional NLRP3 phenotype are associated with the development of CD, suggesting a protective role for NLRP3 inflammasome in the pathogenesis of CD, as shown in Figure 2. Previously, NOD2 and NLRP3 are both belong to the NLRs family, the NLR protein CARD15/NOD2 has been reported to be correlated with CD (75). Remarkably, Villan et al. (76) found that SNPs rs10733113 in the NLRP3 gene region strongly contributed disease susceptibility to CD, although Lewis et al. (77) could not replicate this association in a Large UK Panel. Subsequently, Schoultz et al. (78) have reported that men carrying with both the C10X allele in CARD8, Q705K allele in NALP3, and wild-type alleles of NOD2 demonstrated a disease susceptibility to CD in a cohort of Swedish men, with an obvious sex difference in the genetic susceptibility pattern. Moreover, recent findings suggested that a loss function CARD8 mutation was also showed to activate NLRP3 inflammasome and contribute to the development of CD (79, 80). Lately, polymorphisms of the NLRP3 effector IL-18(rs1946518 A>C, rs360718 A>C, and rs187238 G>C) were reported to be associated with an increased susceptibility to CD (81). Nonetheless, a lately study from China demonstrated that both rs10925019 and rs10754558 could contribute the susceptibility to UC, but not to CD in Han Chinese (82). A recent GWAS meta-analysis has shown that SNPs that affect receptors downstream of NLRP3, such as IL18R1, IL1R1, IL1RL1, IL1RL2, and IL1R2, are associated with susceptibility to IBD (83). In a lately case-control study, SNPs in NLRP3 (rs10754558) have been significantly associated with UC, as “GG” genotype of rs10754558 was 2.48 times more common among UC patients (*P* = 0.04), while “CG” genotype was more frequently found in healthy subjects (84). These results suggest that the polymorphisms of NLRP3 gene may lead to a decrease in the expression of the NLRP3 inflammasome and affect genetic susceptibility to IBD. Nevertheless, IL-18 has been found to be elevated in patients with CD and to play a role in promoting pathogenic T helper 1 (TH1) responses (85). And recent clinical studies also showed an increased expression of proinflammatory cytokines IL-1β secreted from colonic tissues and macrophages of patients with IBD, and the increased IL-1β level is correlated with the diseases severity of IBD (86, 87). Besides, a current study has reported a subtle difference between CD and UC in the activation of NLRP3 inflammasome, which suggested that NLRP3 inflammasome was activated in 60% of CD patients compared to 28.6% of controls (*p* = 0.042) after peripheral blood mononuclear cells stimulation, whereas no significant difference was detected between UC and controls, and among UC patients, NLRP3 activation was associated (*p* = 0.008) with long-standing disease (>1.5 years), implying a differentiation in the UC immunological profile during the progression of the disease (88).

### Animal Studies

Despite no model can perfectly imitate all clinical manifestations and mechanisms of human IBD, various mice models of experimental colitis have been developed to study the mechanisms of human IBD. The dextran sodium sulfate (DSS) model has been extensively used to explore immune mechanisms of colitis. Oral administration of DSS can directly damage the colonic epithelium and trigger inflammation by destroying the compartmentalization of commensal bacteria in the gut. This model exhibits some clinical features including loss of weight, diarrhea, rectal bleeding and even mortality, and the histopathological analysis shows extensive crypt and epithelial cell damage, significant infiltration of macrophages and neutrophils, tissue edema and ulceration (89), which are similar to the pathological findings in IBD patients. Early studies employing mutant mice confirmed that activated caspase-1 was crucial for DSS-induced inflammation, as mice deficient in caspases-1 or NLRP3 experienced significantly less severe pathology than wild-type (WT) mice, which was correlated with reduced levels of IL-1β and IL-18, indicating that excessive production of IL-18 could aggravate the DSS induced colitis (90–92). Several articles have demonstrated that administration of CAI, oroxylin A, or wogonoside could alleviate the severity of experimental colitis, suppress the mucosal inflammation, which might be attributed to its inhibition of NF-κB and NLRP3 inflammasome activation (93, 94). Remarkably, data are claiming that the compound MCC950 can significantly suppress the release of proinflammatory cytokines IL-1β, IL-18, and IL1-α, contribute to inflammatory effects resulting from canonical and non-canonical NLRP3 inflammasome activation in colitis (93, 94). In contrast, recently a number of researches suggested that NLRP3 inflammasome has the function of maintaining gut homeostasis and aiding in protecting from colitis, which have changed past views, suggesting its protective role in intestinal inflammation. Mice deficient in NLRP3, Casp1/11, ASC, and IL-1β have all demonstrated an increased susceptibility to DSS-induced colitis, disease exacerbation and more frequent mortality when compared to WT mice (62, 67, 95–97). Zaki et al. (67) reported that after oral administration of DSS, mice deficient in NLRP3 led to a loss of epithelial integrity, resulting in systemic dispersion of commensal bacteria, massive leukocyte infiltration in the colon and more severe colitis, and indicated that the protective effect of NLRP3 inflammasome on colitis was that it could promote the secretion of IL-18, and an injection of exogenous recombinant IL-18 could partially alleviate the inflammatory symptoms of DSS induced colitis. In addition, Hirota et al. (95) reported that the protective anti-inflammatory cytokines IL-10 and TGF-β decreased in the colon of NLRP3^−/−^ mice, whereas inflammation scores and MPO activity which can reflect macrophage and neutrophils infiltration increased as compared with WT mice, and mice deficient in NLRP3 also showed a decline in the ability to resist microbes, which might be related to decreased B-defensins levels. Meanwhile, once the NLRP3 inflammasome was activated, the production of IL-1 β and IL-18 decreased in mice deficient in NLRP3, further might hinder the repair mechanisms and increase the permeability of intestinal epithelium (89). Noteworthy, one recent study from Japan reported that after the induction of oxazolone-induced colitis (a mouse UC model), NLRP3^−/−^ or Caspase-1^−/−^ mice exhibited a higher sensitivity with an enhanced expression of Th2 cytokine (including IL-4 and IL-13) and a decreased production of mature IL-1β and IL-18 as compared to WT mice, and either exogenous IL-1β or IL-18 ameliorated the colitis (66). Besides, some studied have showed that upon infection with the attaching/effacing intestinal pathogen *Citrobacter rodentium*, mice deficient in NLRP3 and ASC displayed an increased bacterial colonization and dispersion, more severe weight loss and exacerbated intestinal inflammation as compared to WT mice, indicating an early activation of NLRP3 in intestinal epithelial cells could limit pathogen colonization and prevent subsequent pathology and intestinal inflammation, and ascribed this protective effect to the production of cytokines IL-1β and IL-18 (56, 98). These findings reinforce the concept that innate immune recognition at the epithelial barrier has a crucial function in the initiation of protective immunity. Taken together, the consensus seems that NLRP3 inflammasome activation likely triggers beneficial responses following impairment of intestinal epithelial integrity, and then promotes the replacement of damaged enterocytes, which are involved in intestinal tissue repair mechanisms following injury, at least in the initial stage of disease (part 2 of Figure 1).

## NLRP3 Inflammasome and gut Microbiota

More than 500 different species of bacteria live in human gut and the number of bacteria is up to 10^14^ (99), this intestinal microflora serves various roles including metabolic, protective, and immunological functions (100). Indeed, microbiota itself is a non-immune component of mucosal immunity, but it can interact with the immune components of microbial immunity such as immune cells and soluble factors. Dysregulation or alterations in the microbiota composition may be involved in the pathogenesis of IBD (101). In general, UC patients have higher overall bacterial counts than CD patients. The latter, however, exhibits a higher proportion of *unclassified Bacteroidetes* spp. (102). Recent studies have suggested a key role for NLRP3 inflammasome in shaping the composition of the intestinal microbiota. Alterations in the quantity and composition of the microbiota were also observed in NLRP3^−/−^ mice. Initially, it was found that there were more bacteria in the colon of mice deficient in NLRP3 than that of WT mice, and the analysis of colonic bacteria showed that these increased bacteria in mice deficient in NLRP3 belonged to *S. thuringiensis*, including different *Clostridium, Rod bacteria*, and *Proteobacteria* (97). Besides, NLRP3^−/−^ mice with an increased susceptibility to colitis showed that altered B-defensins levels were most likely due to an altered microbiota composition in gut (95). Moreover, oral administration of DSS was thought to be directly toxic to colonic epithelial cells and could trigger intestinal inflammation by disrupting the composition of microbial in gut (103). Additional studies subsequently speculated that the results of different experiments might have been an artifact of the differential constitution of microbiota between the WT and knockout mouse lines. Further support comes from the recent finding by Yao et al. using the gain of function NLRP3 R258W mice and monitoring their microbiota shift. They have dissected a complex crosstalk between NLRP3 inflammasome and gut microbiota. They also found that the hyperactive NLRP3 inflammasome, which led to a local over-production of IL-1β, could maintain gut homeostasis and confer a strong resistance to experimental colitis through a remodeled gut microbiota with an enhanced anti-inflammatory capacity due to an increased induction of regulatory T cells (48). Notably, it is recently reported that intestinal commensal microbes can stimulate excessive or persistent inflammation in genetically susceptible individuals, which sheds light on the elucidation of the etiology of IBD (104).

## Conclusion

A properly mounted immune response is crucial for the body to recognize and eliminate danger, and acute inflammation is often self-limiting and is normally attenuated if stimuli are removed, and then homeostasis can be restored and tissue repair is initiated. But unresolved inflammation may result in chronic autoimmune diseases, such as IBD (105). Population-based studies have identified some potential risk polymorphisms associated with IBD. The dysregulation of NLRP3 inflammasome and its importance in maintaining intestinal health and mucosal immune response have been demonstrated by mice models of colitis. Undeniably, all studies have clearly indicated that NLRP3 inflammasome plays a key role in the pathogenesis of colitis although the results are still controversial. Increasing evidence suggested that its activation could exert an effective response when the intestinal epithelial integrity was impaired, which could promote a repairment and regeneration of the intestinal mucosa, at least in the early stages of the disease. The different results that might depend on variations in mouse/human genetic backgrounds, differences in the composition of the gut microbiota, choice of colitis models or approaches to induce colitis (percentage of DSS, duration of DSS administration and number of cycles) and so on. For instance, NLRP3 inflammasome will manifest as damage factor when 2% DSS is administrated for 9 days, while it shows a protective effect when classic 3–5% DSS is administrated for 5 days. Likewise, different kinds of mice may cause different results too, most studies about NLRP3 inflammasome were made in C57 mice, while some researchers used BALB/C mice, these slightly difference might contribute to different experimental results. And the composition of the intestinal microflora can significantly influence disease severity in IBD models comparing WT and NLRP3^−/−^ mice. This may partially explain contradictory results in different labs. Furthermore, studies of inflammasome in CD and UC are not always consistent (82, 88). Another explanation for the different results is that the activation of NLRP3 inflammasome has a characteristic of location-specificity, Lissner et al. (106) suggested that the activation of NLRP3 inflammasome in intestinal epithelium plays a protective role, as it would maintain homeostasis through regulation of commensal microbiota and eradication of harmful bacterial, and defensin synthesis. However, once the epithelial barrier was disrupted (as occurs in IBD patients and DSS-induced colitis models), the microbiota would infiltrate into the lamina propria and recruit immune cells, in such a case, its activation might well have a deleterious effect on mucosal inflammation. It is thus likely that the activation of NLRP3 inflammasome in IBD leads to two possibilities. First, the inflammasome enhances inflammation, resulting in aggravation of colonic damage. Second, in response to inflammation, the inflammasome ameliorates colitis and then prevents further damage. Further studies are warranted to define the precise role of NLRP3 inflammasome in non-inflamed mucosa under steady state conditions and in IBD. The role of NLRP3 inflammasome in IBD is just beginning to be clarified, much is still unknown, and the differences between animal and human experiments are waiting on further researches. Undoubtedly, a clear understanding of both the basic physiology and precise mechanisms of the NLRP3 inflammasome will guide the development of future effective therapeutics for IBD.

## Author Contributions

YZ and HZ reviewed the literature. YZ wrote the manuscript. HZ supervised this preparation of this review, and edited and approved the manuscript.

### Conflict of Interest Statement

The authors declare that the research was conducted in the absence of any commercial or financial relationships that could be construed as a potential conflict of interest.
